# Relapsed acute lymphoblastic leukemia with unusual multiple bone invasions: A case report

**DOI:** 10.3892/ol.2014.1820

**Published:** 2014-01-23

**Authors:** MAYUMI HANGAI, KENTARO WATANABE, RYOSUKE SHIOZAWA, MITSUTERU HIWATARI, KOHMEI IDA, JUNKO TAKITA

**Affiliations:** Department of Pediatrics, The University of Tokyo Hospital, Tokyo 113-8655, Japan

**Keywords:** acute lymphoblastic leukemia, bone relapse, positron emission tomography, computed tomography

## Abstract

The present study describes a unique pediatric case with multiple bone invasions of acute lymphoblastic leukemia (ALL) during remission. An eight-year-old male with a history of ALL was admitted complaining of intermittent and migrating pain in the limb 2 years following complete remission. Magnetic resonance imaging and whole-body positron emission tomography with ^18^F-fluorodeoxyglucose revealed abnormal multifocal involvement in the bones and corresponding soft tissues. Repeated bone marrow (BM) aspiration indicated normal cellular marrow without leukemic cells, and marked leukemic cell infiltration in different sections of the ilium, respectively. These findings suggested isolated bone relapse, and it is probable that systematic BM relapse occurred as a consequence.

## Introduction

Acute lymphoblastic leukemia (ALL) is the most prevalent hematological malignancy in children (1). Although remission is achieved in the majority of children with ALL using modern therapies, disease relapse occurs in 15–20% of patients (2). The greatest number of relapses occur in the bone marrow (BM), in an isolated form or combined with involvement of another site, mainly the central nervous system (CNS) or testes. Isolated CNS or testicular relapse or, less frequently, relapse involving other extramedullary sites, may also occur. Isolated extramedullary relapse in childhood ALL is associated with a wide variety of clinical symptoms and often presents a diagnostic challenge (2). The current report presents a case of relapsed ALL with unusual intermittent and migrating bone pain caused by multiple bone invasions prior to clinical manifestation of BM relapse of ALL.

## Case report

**Figure 1:**
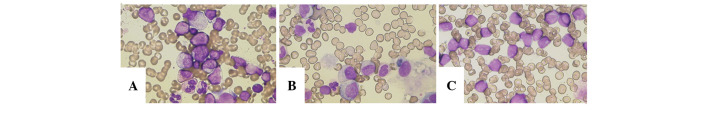
May-Grünwald Giemsa stain of BM aspirates. (A) BM aspiration from the left ilium showing lymphoid hyperplasia with 52.2% blasts. (B) BM aspiration from the anterior left ilium revealing no monoclonal blasts on the basis of morphology and cell surface markers. (C) Subsequent BM aspiration from the posterior left ilium showing infiltration of monotonous blast cells. BM, bone marrow.

An eight-year-old male was admitted to the University of Tokyo Hospital (Tokyo, Japan) with a history of intermittent and migrating limb pain, claudication and a 9-month fever. Repeated laboratory examinations during that period revealed no hematological abnormalities. Patient history included diagnosis of ALL at the age of 5 years, and remission for 2 years prior to admission. Physical examination revealed no pallor, lymphadenopathy, organomegaly or petechiae. Laboratory studies revealed normal blood cell counts. Serum calcium and alkaline phosphatase were also within the normal range. C-reactive protein levels were slightly elevated (2.61 mg/dl) and blood cultures were negative. Soluble interleukin 2 receptor levels were elevated to 960 U/ml (127–582 U/ml: normal range). BM aspiration of the anterior left ilium revealed lymphoid hyperplasia with 52.2% blast-like cells (Fig. 1A). However, flow cytometry was not performed at this time, and thus, diagnosis of relapse could not be confirmed. Therefore, a second BM aspiration from the posterior left ilium was performed, but no monoclonal blasts were evident on the basis of morphological and cell surface marker analyses (Fig. 1B).

**Figure 2:**
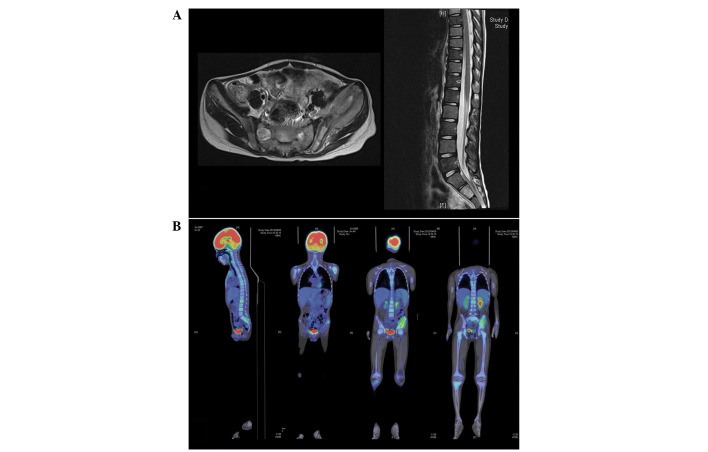
Radiographic images at the time of relapse. (A) T2-weighted magnetic resonance image of the abdomen and vertebrae. Infiltrative process in the ilium, adjacent soft tissue and multiple vertebral bodies are indicated. (B) ^18^F-fluorodeoxyglucose positron emission tomography showing hypermetabolic foci in the left ilium, the epiphysis of the left humerus, the proximal end of the right tibia and multiple vertebral bodies corresponding to areas of marrow infiltration.

Abdominal computed tomography (CT) revealed lysis and destruction of the left ilium. Magnetic resonance imaging (MRI) revealed infiltrative processes in the ilium, the adjacent soft tissue and multiple vertebral bodies (Fig. 2A). Whole-body positron emission tomography (PET) with ^18^F-fluorodeoxyglucose (FDG) revealed hypermetabolic foci in the left ilium, the epiphysis of the left humerus, the proximal end of the right tibia and multiple vertebral bodies corresponding to the areas of marrow infiltration visualized on MRI (Fig. 2B). These radiographic findings suggested malignancy; thus, CT-guided biopsies were performed. Pathologically, cluster of differentiation (CD) 10, CD20, CD79a and terminal deoxynucleotidyl transferase (TdT) antigens indicated atypical lymphocytes infiltrating the left ilium and adjacent soft tissue.

Subsequent BM aspiration from the anterior and posterior left ilium revealed infiltration of monotonous blast cells (anterior, 71.6%; posterior, 99.6%) (Fig. 1C). Immunophenotyping identified expression of CD10, CD19, CD22, CD24, cytoplasmic (cy) CD22, cyCD79a, cy-TdT, human leukocyte antigen-DR, CD34, CD99, CD38 and CD58 antigens in the blasts. Taken together, these findings suggested a diagnosis of isolated extramedullary bone relapse and subsequent BM relapse of B-precursor ALL. Chemotherapy following the Japanese Pediatric Leukemia/Lymphoma Study Group Protocol, ALL-R08, for S2-risk ALL (vincristine, methotrexate, L-asparaginase, cytarabine, 6-mercaptopurine, vindesine, ifosfamide and daunorubicin), induced complete clinical and cytogenetic remission (unpublished data). This study was approved by the ethics committee of the University of Tokyo, Tokyo, Japan (no. 2701). Written informed consent was obtained from the patient’s family.

## Discussion

The patient described in the current study presented with severe intermittent and migrating bone pain of long duration, claudication and fever, but no hematological abnormalities. These findings suggested that isolated bone relapse had developed prior to clinical manifestation of BM relapse of ALL. The radiographic findings showed multifocal invasion of the bone and surrounding soft tissues, which also corresponded with the possibility of initial isolated bone tissue invasions.

Notably, repeated BM aspiration in different sections of the ilium revealed no diffuse homogenous involvement in the BM. This suggested that the leukemic cells had infiltrated the BM in a localized manner mimicking a solid tumor at the time of initial BM relapse. Extramedullary tissue infiltration as a solid tumor is a common feature of a subset of acute myeloid leukemia (3), but is rarely observed in ALL. In addition, isolated bone relapse occurring during complete BM remission in childhood ALL is less common (4). Furthermore, localized ALL relapse in BM is extremely rare (5). Therefore, these features could cause diagnostic difficulty.

FDG-PET has been widely used in the evaluation of various malignancies, but clinical application in cases with leukemia remains limited (6). Thus, the present case demonstrates the diagnostic value of FDG-PET for detection of focal localization in leukemia.

The differential diagnoses of cases with multiple bone invasions are as follows: Metastases of solid tumor, osteosarcoma, Langerhans cell histiocytosis, osseous lymphoma, bacterial osteomyelitis, chronic recurrent multifocal osteomyelitis (CRMO) and autoinflammatory disease. CRMO is one of the most important differential diagnoses of multiple bone invasions due to its similarity in radiographic imaging to our case (7). Although morphologically CRMO is a reactive condition (8), cases of CRMO following ALL (9) and osseous lymphoma following CRMO (10) have been reported. It was hypothesized that common genetic factors may predispose patients with ALL to CRMO and lymphoblastic proliferations.

In the present case, CRMO may have been an alternative diagnosis based on the radiographic images, but a patient history of ALL suggested the possibility of relapse. BM aspiration or other tissue biopsy is necessary, but not sufficient, to rule out suspected malignancy. Relapsed or primary hematological neoplasms must not be excluded based on the results of a single BM aspiration; radiographic imaging must also be utilized in the differential diagnosis of cases with multiple bone lesions, even in the presence of apparently normal blood cell counts.

Notably, no bone metastasis was detected in the initial episode of ALL in the present case. At the time of relapse, the leukemic cells may have infiltrated multiple bone and soft tissue sites mimicking lymphoma or other solid tumors without hematological changes in peripheral blood. This suggests that the leukemic cells acquired affinity to the bone rather than the BM. The molecular characteristics of the primary and relapsed leukemic cells are likely to have differed. Since identifying the molecular features of relapsed clones may shift the therapeutic target, evaluating the molecular differences between primary and relapsed leukemic cells is important not only biologically but also clinically. Further investigation will improve the understanding of the mechanisms involved in differentiation and clonal evolution of leukemic cells.

In conclusion, isolated bone relapse in childhood ALL is uncommon. The use of imaging investigation may be useful if a patient in remission develops bone pain without presenting with abnormal findings of BM aspiration, in order to assess whether the patient has relapsed. Our report provides an improved understanding of the development and diagnosis of isolated bone relapse in childhood ALL
